# Communication Competence and Nurse Managers’ Authentic Leadership in Relation to Nursing Performance Among Operating Room Nurses: The Role of Teamwork in a Cross-Sectional Study

**DOI:** 10.3390/healthcare14213706

**Published:** 2026-11-01

**Authors:** Jin Seon Park, Hyun-Ju Lee

**Affiliations:** 1 Regional Trauma Center, Pusan National University Hospital, 179 Gudeok-ro, Seo-gu, Busan 49241, Republic of Korea; wlstjs4707@pnuh.co.kr; 2 College of Nursing, Catholic University of Pusan, 57 Oryundae-ro, Geumjung-gu, Busan 46252, Republic of Korea

**Keywords:** nurses, perioperative nursing, communication, authentic leadership, teamwork, work performance

## Abstract

**Highlights:**

**What are the main findings?**

• Communication competence and nurses’ perceptions of nurse managers’ authentic leadership were each positively associated with nursing performance among OR nurses, and their direct associations with nursing performance remained significant across model specifications.
• Neither indirect effect remained statistically significant in the mutually adjusted model using the full teamwork measure, whereas both were significant in the sensitivity analyses excluding the T-TPQ leadership subscale, with or without the communication subscale. These findings indicate that the indirect associations were sensitive to model specification and the measurement of teamwork.

**What are the implications of the main findings?**

• Strategies to support nursing performance among OR nurses may consider approaches focused on individual nurses, nurse managers, and teams, rather than focusing on competence or leadership alone.
• These findings highlight the relevance of considering perceived teamwork alongside nurses’ communication competence and their perceptions of nurse managers’ authentic leadership when exploring approaches to support nursing performance among OR nurses.

**Abstract:**

**Background/Objectives:** Nursing performance in the operating room (OR) is associated with individual competencies, leadership, and teamwork. Based on McGrath’s Input–Process–Output model, this study examined the associations of OR nurses’ communication competence and nurses’ perceptions of nurse managers’ authentic leadership with nursing performance including indirect associations through teamwork. **Methods:** This cross-sectional study included 149 OR nurses working at three tertiary hospitals in Republic of Korea. Self-reported data were analyzed using PROCESS macro Model 4 with 5000 bootstrap resamples and 95% percentile bootstrap confidence intervals. Separate mediation models were supplemented by mutually adjusted models including both predictors and sensitivity analyses with modified teamwork scores. **Results:** OR nurses’ communication competence and nurses’ perceptions of nurse managers’ authentic leadership were each positively associated with teamwork and nursing performance. Their direct associations with nursing performance remained statistically significant across model specifications. Indirect associations through teamwork were significant in the separate models but not in the mutually adjusted model using the full teamwork measure. Both indirect associations were significant when the T-TPQ leadership subscale was excluded, with or without the communication subscale, although their estimates were smaller. **Conclusions:** Direct associations with nursing performance were consistent, whereas indirect associations through teamwork depended on model specification and teamwork measurement. These findings support considering teamwork alongside OR nurses’ communication competence and nurses’ perceptions of nurse managers’ authentic leadership without implying causal relationships.

## 1. Introduction

The operating room (OR) is a high-risk, highly complex clinical environment in which multidisciplinary professionals, including surgeons, anesthesiologists, and nurses, must work closely together to perform complex surgical procedures within a limited timeframe [1,2]. During surgery, patients’ conditions and the course of surgical procedures can change rapidly, and the high degree of interdependence among team members requires not only accurate task performance but also timely information exchange and coordination [3,4]. In particular, OR nurses contribute to safe and efficient surgical care by preparing surgical instruments and equipment, maintaining aseptic technique, ensuring patient safety, and collaborating with members of the surgical team [5,6,7]. Effective performance of these roles is reflected in OR nurses’ nursing performance, an important aspect of nursing practice associated with patient safety, surgical outcomes, and healthcare quality [8,9,10]. Therefore, identifying factors associated with nursing performance among OR nurses is important for supporting safe, high-quality perioperative nursing care.

Nursing performance among OR nurses is associated not only with individual nurses’ competencies but also with collaboration and interactions among team members. Previous studies have reported that, in addition to individual and job-related characteristics, relational factors such as collaboration among colleagues are associated with nursing performance and the quality of perioperative nursing care [9,11,12]. Therefore, understanding nursing performance in the OR requires a broader consideration of factors related to individual nurses, nurse managers, and interactions among team members.

One individual competency that may be particularly relevant to nursing performance among OR nurses is communication competence. OR nurses continuously exchange information and collaborate with various healthcare professionals while performing their duties; therefore, the ability to communicate effectively and accurately is considered an essential competency in OR nursing practice [13]. Previous research involving hospital nurses has identified a positive association between communication competence and nursing performance [14,15], and a recent study involving OR nurses reported a positive correlation between communication competence and teamwork [16]. Taken together, these findings suggest that communication competence should be considered not only as an individual competency associated with nursing performance among OR nurses but also as an individual capability that may be relevant to collaborative interactions among team members.

In addition to nurses’ individual competencies, nurse managers’ leadership may also be relevant to team interactions and nursing performance. Authentic leadership encompasses self-awareness, relational transparency, balanced processing, and an internalized moral perspective, which underpin leaders’ relationships with team members [17]. Previous studies have reported that nurse managers’ authentic leadership is significantly associated with collaboration and teamwork competence among team members [18,19,20], as well as with nurses’ work-related performance, and quality of care [21,22,23]. These findings provide a basis for considering teamwork when examining the association between nurse managers’ authentic leadership and nursing performance among OR nurses.

Teamwork is an interactive process in which team members communicate, coordinate roles and resources, and collaborate to achieve shared goals [24]. It is particularly important in the OR, where tasks are highly interdependent [24,25]. Teamwork involves information sharing and task coordination among surgical team members, and previous studies have shown that teamwork in perioperative nursing settings is associated with nursing practice and patient safety-related outcomes [11,26,27,28]. A recent systematic review also reported that nurses’ positive perceptions of teamwork were associated with favorable nurse- and patient-related outcomes, including patient safety competencies and the quality of nursing care [29]. These findings provide a basis for examining teamwork in relation to OR nurses’ communication competence, their perceptions of nurse managers’ authentic leadership, and nursing performance.

Existing evidence indicates that nurses’ communication competence [14,15], nurse managers’ authentic leadership [21,22], and teamwork [26,27] are each associated with nursing performance or nursing-related outcomes. However, previous research has primarily examined these relationships separately, and limited attention has been given to whether individual competencies and leadership-related factors are associated with nursing performance through a common team interaction process. In particular, evidence regarding these relationships among OR nurses remains limited. Examining communication competence and nurses’ perceptions of nurse managers’ authentic leadership as conceptually distinct factors in relation to a common teamwork process may extend existing evidence by considering both their direct associations with nursing performance and their indirect associations through teamwork, thereby providing a more integrated understanding of nursing performance in the highly interdependent OR context. Accordingly, this study aimed to examine the associations of OR nurses’ communication competence and their perceptions of nurse managers’ authentic leadership with nursing performance and whether these associations included significant indirect associations through teamwork.

### 1.1. Theoretical Framework

This study applied McGrath’s Input–Process–Output (IPO) model as the theoretical framework for conceptualizing the relationships among the study variables [30]. The IPO model explains team functioning and performance through a structure in which Inputs, as antecedent factors that shape team functioning, are linked to Outputs through Processes, which represent interactions among team members as they perform their tasks [30,31]. Inputs include individual characteristics, such as team members’ knowledge, skills, and attitudes, as well as team- and contextual-level factors such as leadership, whereas Processes include team interactions such as communication, coordination, and collaboration [31]. Previous healthcare research has similarly applied the IPO framework to conceptualize teamwork as a team interaction process associated with clinical performance [32] and to examine its mediating role in the relationship between nursing team roles and team performance [33]. Although the IPO model was originally developed to explain team-level functioning and performance, the present study used it as a heuristic framework to organize hypothesized associations among individually measured variables. Thus, teamwork was assessed through individual nurses’ perceptions, and nursing performance was assessed through individual self-reports, without aggregating these measures to the team level.

### 1.2. Research Hypotheses

**Figure 1:**
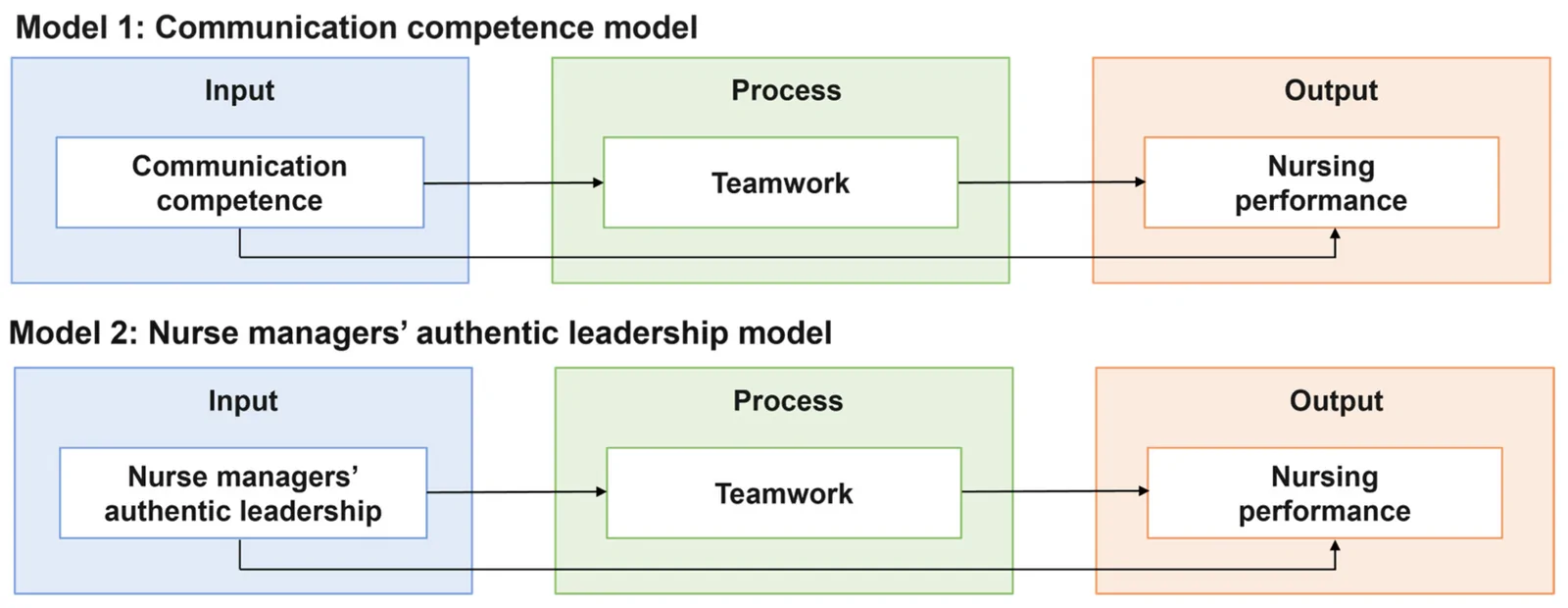
Conceptual framework of the two hypothesized models of this study. Note. The two models were examined separately. Nurse managers’ authentic leadership refers to nurses’ perceptions of nurse managers’ authentic leadership. The arrows represent hypothesized associations and do not imply causal or temporal relationships.

Based on this IPO framework, nurses’ communication competence was conceptualized as an individual-level Input, nurses’ perceptions of nurse managers’ authentic leadership as a leadership-related Input, teamwork as the Process, and nursing performance as the Output (Figure 1). These two Inputs represent conceptually distinct factors, namely individual communication competence and perceived managerial leadership. Both may be associated with perceived teamwork and nursing performance. Accordingly, two sets of hypothesized associations were examined separately, with teamwork as the common Process.

The following hypotheses were formulated based on these proposed relationships.

**Hypothesis** **1.** *Communication competence is positively associated with teamwork.*

**Hypothesis** **2.** *Communication competence is positively associated with nursing performance after controlling for teamwork.*

**Hypothesis** **3.** *Teamwork is positively associated with nursing performance.*

**Hypothesis** **4.** *Communication competence has a significant indirect association with nursing performance through teamwork.*

**Hypothesis** **5.** *Nurses’ perceptions of nurse managers’ authentic leadership are positively associated with teamwork.*

**Hypothesis** **6.** *Nurses’ perceptions of nurse managers’ authentic leadership are positively associated with nursing performance after controlling for teamwork.*

**Hypothesis** **7.** *Nurses’ perceptions of nurse managers’ authentic leadership have a significant indirect association with nursing performance through teamwork.*

## 2. Materials and Methods

### 2.1. Study Design and Setting

This cross-sectional correlational study examined the indirect role of teamwork in the associations of OR nurses’ communication competence and their perceptions of nurse managers’ authentic leadership with nursing performance. Participants were recruited from the ORs of three tertiary hospitals located in Busan, Republic of Korea. Each hospital had approximately 800–1200 beds and performed approximately 11,000–17,000 surgical procedures annually in 2025. The operating rooms supported surgical procedures across a range of specialties. Each hospital had approximately two to three operating room nurse managers. The study was reported in accordance with the Strengthening the Reporting of Observational Studies in Epidemiology (STROBE) guidelines for cross-sectional studies [34].

### 2.2. Participants and Sample Size

Participants were recruited using convenience sampling. Participants were eligible if they were currently working in an OR as a scrub or circulating nurse and had at least one year of perioperative nursing experience. Nurses with less than one year of perioperative nursing experience and nurse managers who were not directly involved in perioperative nursing care were excluded. Approximately 240 operating room nurses across the three participating hospitals (approximately 90, 80, and 70 nurses, respectively) met the study eligibility criteria and constituted the eligible recruitment pool.

The required sample size was estimated using G*Power version 3.1.9 for multiple regression analysis [35]. The calculation included 14 potential predictors, comprising three main study variables and 11 demographic and work-related characteristics, a medium effect size (f^2^ = 0.15) based on Cohen’s criteria [36], with reference to the sample size calculation approaches reported in previous nursing studies [9,37]. With a significance level (α) of 0.05 and statistical power (1 − β) of 0.80, the minimum required sample size was 135. To account for an anticipated 10% rate of nonresponse or incomplete questionnaires, the target sample size was set at 150. A total of 150 questionnaires were distributed across the three hospitals (60, 50, and 40 questionnaires, respectively), considering the relative size of their OR nursing workforce. All 150 questionnaires were returned, yielding a questionnaire return rate of 100%. The 150 participants represented approximately 62.5% of the estimated eligible recruitment pool of 240 nurses. One questionnaire was excluded due to missing responses, resulting in a final sample of 149 nurses (59, 50, and 40 nurses from the three hospitals, respectively).

### 2.3. Measures

#### 2.3.1. Demographic and Work-Related Characteristics

Demographic and work-related characteristics were assessed using a self-administered questionnaire. Demographic characteristics included age, gender, marital status, religion, and education level. Age was recorded in completed years. Gender was assessed as male or female; marital status as single, married, or other; religion as having or not having a religion; and education level as diploma, bachelor’s degree, or master’s degree or higher. Work-related characteristics included job position, total clinical experience, perioperative nursing experience, work schedule, reason for OR assignment, and job satisfaction. Job position was assessed as staff nurse, charge nurse, or other. Total clinical experience and perioperative nursing experience were recorded in years and months. Work schedule was assessed as fixed daytime work, three-shift rotation, or two-shift rotation; reason for OR assignment as personal preference, hospital assignment, or other; and job satisfaction as very satisfied, satisfied, neutral, dissatisfied, or very dissatisfied. No participants selected the ‘other’ category for marital status, job position, or reason for OR assignment. For analysis, three-shift and two-shift rotations were combined into the shift-work category, and job satisfaction was categorized as satisfied (very satisfied or satisfied), neutral, or dissatisfied (dissatisfied or very dissatisfied).

#### 2.3.2. Communication Competence

Nurses’ communication competence was measured using the Global Interpersonal Communication Competence (GICC), originally developed by Hur [38] and subsequently adapted for clinical nurses by Lee and Kim [39]. The instrument consists of 15 items, each rated on a 5-point Likert scale ranging from 1 (“Not at all”) to 5 (“Very much”), with higher scores indicating greater communication competence. The Cronbach’s α values were 0.72 in the original instrument development study [38], 0.83 in the study by Lee and Kim [39], and 0.82 in the present study.

#### 2.3.3. Nurses’ Perceptions of Nurse Managers’ Authentic Leadership

Nurses’ perceptions of nurse managers’ authentic leadership were measured using the Authentic Leadership Questionnaire (ALQ), originally developed by Avolio et al. [40], validated by Walumbwa et al. [17], and subsequently adapted for nursing organizations by Song and Seomun [41]. Permission to use the ALQ was obtained from Mind Garden, the copyright holder. Participants were instructed to evaluate their immediate nurse manager when responding to both the authentic leadership measure and the leadership subscale of the teamwork measure. The instrument consists of 16 items across four subscales: self-awareness (4 items), relational transparency (5 items), internalized moral perspective (4 items), and balanced processing (3 items). Each item was rated on a 5-point Likert scale ranging from 1 (“Not at all”) to 5 (“Frequently, if not always”), with higher scores indicating higher levels of nurse managers’ authentic leadership as perceived by nurses. In the original validation study, Cronbach’s α coefficients for the four subscales ranged from 0.70 to 0.92 across different samples [17]. Cronbach’s α for the overall scale was 0.96 in the study by Song and Seomun [41]. In the present study, Cronbach’s α was 0.93 for the overall scale, with values ranging from 0.80 to 0.91 across the four subscales.

#### 2.3.4. Teamwork

Teamwork was measured using the Team Strategies and Tools to Enhance Performance and Patient Safety (TeamSTEPPS^®^) Teamwork Perceptions Questionnaire (T-TPQ), originally developed by Battles and King [42] and adapted by Ahn and Lee [43] for healthcare professionals working in ORs to reflect the Korean OR context. The instrument consists of five subscales: team structure, leadership, situation monitoring, mutual support, and communication. Each subscale contains 7 items, for a total of 35 items. Each item is rated on a 5-point Likert scale ranging from 1 (“Strongly disagree”) to 5 (“Strongly agree”), with higher scores indicating greater perceived teamwork. The Cronbach’s α values for the subscales of the original instrument ranged from 0.88 to 0.95 [42]. In the present study, Cronbach’s α was 0.95 for the overall 35-item scale, with values ranging from 0.83 to 0.89 across the five subscales. Cronbach’s α was also 0.95 for the 28-item scale excluding the seven leadership items used in the sensitivity analysis.

#### 2.3.5. Nursing Performance

Nursing performance was measured using the Performance Measurement Scale for Hospital Nurses, originally developed by Ko et al. [44] and modified and supplemented for OR nurses by Moon and Shin [45]. The instrument consists of 17 items across four subscales: performance capability (7 items), performance attitude (4 items), performance improvement (3 items), and nursing process application (3 items). Each item is rated on a 4-point Likert scale ranging from 1 (“Not at all”) to 4 (“Very much”), with higher scores indicating higher levels of nursing performance. The Cronbach’s α value was 0.92 in the original instrument development study [44], and 0.94 in the study by Moon and Shin [45]. In the present study, Cronbach’s α was 0.90 for the overall scale, with values ranging from 0.74 to 0.86 across the four subscales.

### 2.4. Data Collection

Data were collected from 7 April to 18 April 2025. Because direct access to the OR was restricted, the researcher explained the study purpose, eligibility criteria, and data collection procedures to designated nursing administration staff at each hospital and obtained their agreement to cooperate with the study. The number of questionnaires allocated to each hospital was determined in advance in consultation with these staff members, considering the size of the OR nursing workforce at each hospital. The researcher then provided the staff with the study information sheets, informed consent forms, questionnaires, and separate return envelopes. The staff posted recruitment notices in the OR nurses’ changing rooms and placed the study information sheets, informed consent forms, questionnaires, and return envelopes at designated locations, allowing eligible nurses to review the study information and voluntarily decide whether to participate. Eligible nurses participated on a first-come, first-served basis, within the predetermined number of questionnaires allocated to each hospital.

Participants provided written informed consent before completing the questionnaire, which required approximately 10 min. To maintain confidentiality and reduce the potential for evaluation-related response bias, participants were instructed to place their completed questionnaires and signed consent forms in separate envelopes and seal each envelope individually. The sealed envelopes were collected by the designated nursing administration staff and subsequently returned to the researcher.

### 2.5. Data Analysis

Data were analyzed using IBM SPSS Statistics version 27.0 (IBM Corp., Armonk, NY, USA) and PROCESS macro version 5.0 for SPSS. Additional analyses of discriminant validity and common method bias were performed using R software 4.6.1 (R Foundation for Statistical Computing, Vienna, Austria) with the lavaan package (version 0.6-21).

Categorical variables were summarized using frequencies and percentages, and continuous variables were summarized using means and standard deviations. Total clinical experience and perioperative nursing experience were categorized into 1 to less than 5 years, 5 to less than 10 years, and 10 years or more, consistent with previous studies of operating room nurses [5,9]. Internal consistency was assessed using Cronbach’s α coefficients. Differences in the main study variables according to participants’ characteristics were analyzed using independent *t*-tests and one-way ANOVA with Scheffé post hoc tests, and correlations among the main study variables were examined using Pearson’s correlation coefficients.

PROCESS macro Model 4 was applied separately to the two models to estimate the indirect effects through teamwork in the associations of nurses’ communication competence and nurses’ perceptions of nurse managers’ authentic leadership with nursing performance. Education level, job position, and total clinical experience were included as covariates in both models based on their significant associations with nursing performance in the univariate analyses and their relevance to nursing performance reported in previous studies [9,46]. Indirect effects were estimated using 5000 bootstrap resamples, and 95% percentile bootstrap confidence intervals (CIs) were used to evaluate their statistical significance. An indirect effect was considered statistically significant when the CI excluded zero.

To account for the shared variance between the two predictors, additional mutually adjusted mediation analyses were conducted using PROCESS Model 4, with each predictor alternately specified as the independent variable and the other included as a covariate. These analyses initially used the full 35-item T-TPQ teamwork score. To assess the potential influence of measurement overlap between nurses’ perceptions of nurse managers’ authentic leadership and the T-TPQ leadership subscale, a further sensitivity analysis was conducted using a modified 28-item teamwork score excluding the seven-item leadership subscale. The same mutually adjusted model structure and covariates were retained. An additional sensitivity analysis was conducted using a 21-item teamwork score excluding both the leadership and communication subscales of the T-TPQ, while retaining the same mutually adjusted model structure and covariates.

To assess potential overlap between conceptually related constructs, discriminant validity was examined using the heterotrait–monotrait ratio (HTMT) and confidence intervals for latent factor correlations derived from confirmatory factor analysis (CFA). Two separate item-level, two-factor CFA models were estimated using robust maximum likelihood (MLR), and 95% confidence intervals for latent factor correlations were calculated using robust standard errors and the Wald method. Potential common method bias arising from the cross-sectional self-report design was assessed using Harman’s single-factor test and a comparison of single-factor and four-factor measurement models. The measurement models used item parcels for communication competence and subscale scores for the other three constructs, with maximum likelihood (ML) estimation.

Monte Carlo power analyses for the indirect effects were conducted using the observed standardized path coefficients from the two separate mediation models and the mutually adjusted models using the 35-item and 28-item teamwork scores. The analyses were performed using the Monte Carlo Power Analysis for Indirect Effects application developed by Schoemann et al. [47], with a sample size of 149, 5000 replications, and a 95% confidence level.

### 2.6. Ethical Considerations

The study was conducted after approval was obtained from the Institutional Review Board of Catholic University of Pusan (IRB No. CUPIRB-2025-007). Before participation, nurses received information about the study purpose and procedures, the voluntary nature of participation, the right to withdraw without disadvantage, measures to ensure confidentiality and anonymity, and procedures for data storage and disposal. Written informed consent was obtained from all participants before they completed the questionnaire. Participants who completed the questionnaire received a gift worth approximately KRW 5000 as a token of appreciation.

## 3. Results

### 3.1. Differences in Nursing Performance According to Participants’ Demographic and Work-Related Characteristics

The demographic and work-related characteristics of the participants are presented in Table 1. Most participants were women (87.2%), with a mean age of 32.93 ± 7.35 years; the largest age group was 26–30 years (36.9%). Overall, 64.4% were unmarried, 69.8% held a bachelor’s degree, and 62.4% were staff nurses. The mean total clinical experience was 9.50 ± 7.08 years, and the mean perioperative nursing experience was 9.01 ± 6.94 years. Most participants (89.3%) worked shifts. Regarding the reason for OR assignment, 71.1% reported that their assignment was voluntary, and 47.0% reported being satisfied with working in the OR.

Nursing performance differed significantly according to education level, job position, and total clinical experience. Post hoc comparisons showed that nursing performance was higher among participants with a master’s degree or higher educational qualifications than among those with a diploma or a bachelor’s degree (F = 3.22, *p* = 0.043). Regarding job position, nursing performance was higher among charge nurses than among staff nurses (t = −2.74, *p* = 0.007). Regarding total clinical experience, nursing performance was higher among participants with at least five years of total clinical experience than among those with less than five years of total clinical experience (F = 4.14, *p* = 0.018).

### 3.2. Levels of Communication Competence, Nurses’ Perceptions of Nurse Managers’ Authentic Leadership, Teamwork, and Nursing Performance

The descriptive statistics for nurses’ communication competence, nurses’ perceptions of nurse managers’ authentic leadership, teamwork, and nursing performance are presented in Table 2. The mean score for nurses’ communication competence was 3.81 ± 0.40 out of 5. The mean score for nurses’ perceptions of nurse managers’ authentic leadership was 3.86 ± 0.55 out of 5, with self-awareness having the highest subscale score (3.92 ± 0.60) and balanced processing the lowest (3.72 ± 0.68). The mean score for teamwork was 3.80 ± 0.49 out of 5, with situation monitoring having the highest subscale score (3.95 ± 0.58) and leadership the lowest (3.58 ± 0.64). The mean score for nursing performance was 3.30 ± 0.34 out of 4, with performance capability having the highest subscale score (3.37 ± 0.37) and performance attitude the lowest (3.22 ± 0.44).

### 3.3. Correlations Among Communication Competence, Nurses’ Perceptions of Nurse Managers’ Authentic Leadership, Teamwork, and Nursing Performance

The correlations among communication competence, nurses’ perceptions of nurse managers’ authentic leadership, teamwork, and nursing performance are presented in Table 3. Communication competence was significantly and positively correlated with nurse managers’ authentic leadership (r = 0.332, *p* < 0.001), teamwork (r = 0.340, *p* < 0.001), and nursing performance (r = 0.451, *p* < 0.001). Nurse managers’ authentic leadership was significantly and positively correlated with teamwork (r = 0.650, *p* < 0.001) and nursing performance (r = 0.393, *p* < 0.001). Teamwork was also significantly and positively correlated with nursing performance (r = 0.396, *p* < 0.001).

Given that all study variables were measured using self-reported questionnaires completed by the same respondents, potential common method bias was assessed. Harman’s single-factor test showed that the first factor accounted for 25.6% of the total variance. The single-factor measurement model showed poor fit (CFI = 0.589, TLI = 0.526, RMSEA = 0.195, SRMR = 0.135), whereas the four-factor measurement model demonstrated better fit (CFI = 0.905, TLI = 0.880, RMSEA = 0.098, SRMR = 0.069). In addition, the single-factor measurement model showed significantly poorer fit than the four-factor measurement model (Δχ^2^ (6) = 378.27, *p* < 0.001). These findings did not indicate the presence of a dominant single common factor.

### 3.4. Direct and Indirect Associations of Communication Competence and Nurses’ Perceptions of Nurse Managers’ Authentic Leadership with Nursing Performance Through Teamwork

**Figure 2:**
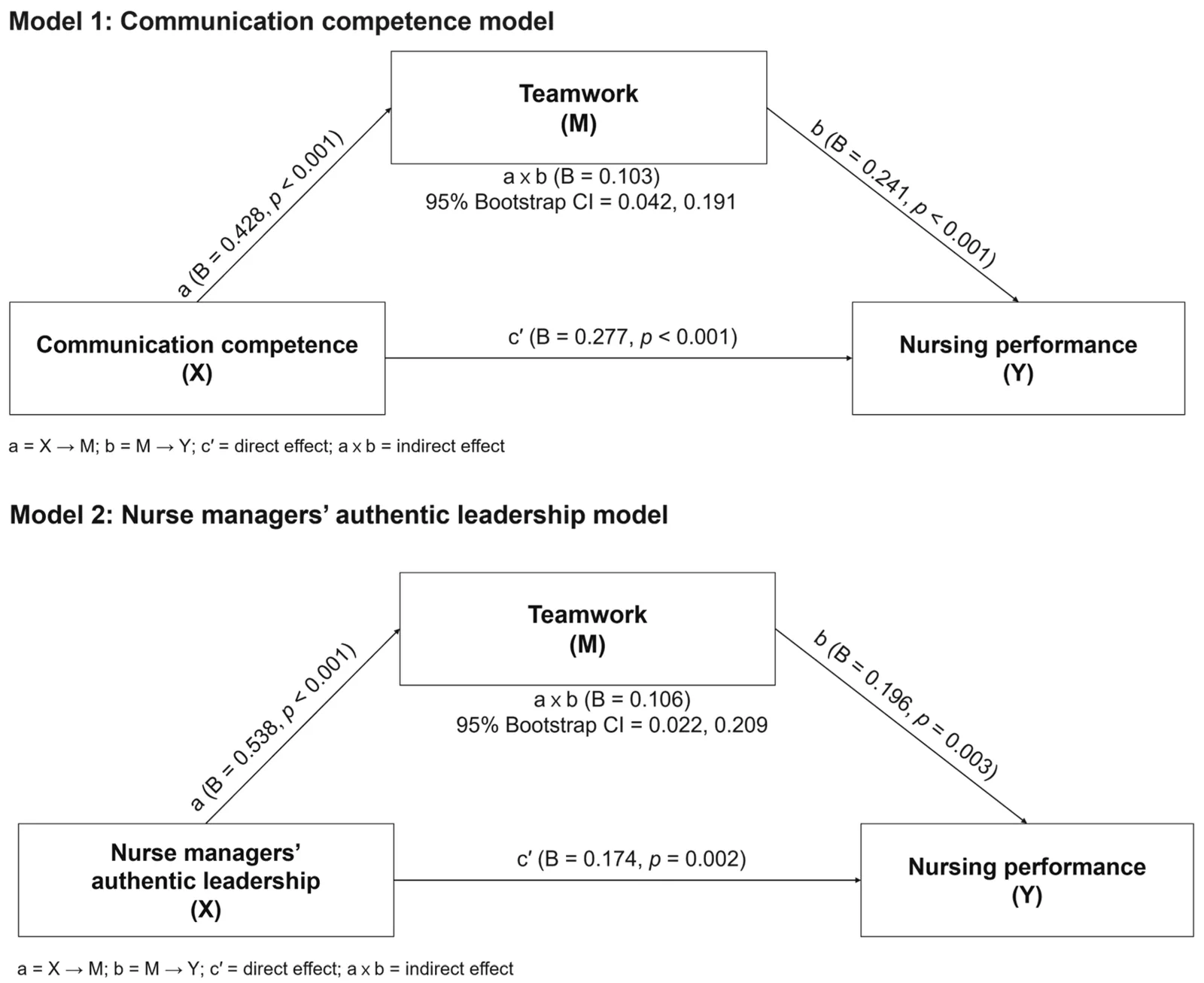
Direct and indirect associations with nursing performance through teamwork. Note. B = unstandardized regression coefficient; CI = confidence interval. The two mediation models were estimated separately and adjusted for education level, job position, and total clinical experience. The 95% percentile bootstrap CIs for indirect effects were estimated using 5000 bootstrap samples. Nurse managers’ authentic leadership refers to nurses’ perceptions of nurse managers’ authentic leadership.

As shown in Table 4 and Figure 2, two separate models were examined. In the communication competence model, communication competence was significantly and positively associated with teamwork (B = 0.428, *p* < 0.001). Communication competence remained significantly and positively associated with nursing performance in the model that included teamwork (B = 0.277, *p* < 0.001). Teamwork was also significantly and positively associated with nursing performance (B = 0.241, *p* < 0.001). The total effect of communication competence on nursing performance was significant (B = 0.380, *p* < 0.001). The indirect effect through teamwork was 0.103 (95% CI [0.042, 0.191]), and the completely standardized indirect effect was 0.122 (95% CI [0.051, 0.222]). Because both confidence intervals excluded zero, the indirect association was statistically significant.

In the authentic leadership model, nurses’ perceptions of nurse managers’ authentic leadership were significantly and positively associated with teamwork (B = 0.538, *p* < 0.001). Nurses’ perceptions of nurse managers’ authentic leadership remained significantly and positively associated with nursing performance in the model that included teamwork (B = 0.174, *p* = 0.002). Teamwork was also significantly and positively associated with nursing performance (B = 0.196, *p* = 0.003). The total effect of nurses’ perceptions of nurse managers’ authentic leadership on nursing performance was significant (B = 0.279, *p* < 0.001). The indirect effect through teamwork was 0.106 (95% CI [0.022, 0.209]), and the completely standardized indirect effect was 0.172 (95% CI [0.036, 0.335]). Because both confidence intervals excluded zero, the indirect association was statistically significant.

### 3.5. Sensitivity and Power Analyses of the Indirect Associations Through Teamwork

To examine the sensitivity of the findings from the separate mediation models to model specification, additional analyses were conducted by simultaneously including communication competence and nurses’ perceptions of nurse managers’ authentic leadership as predictors while retaining the same covariates (Table 5). In the mutually adjusted model using the full 35-item T-TPQ, communication competence (B = 0.202, *p* = 0.015) and nurses’ perceptions of nurse managers’ authentic leadership (B = 0.490, *p* < 0.001) were positively associated with teamwork. Both communication competence (B = 0.253, *p* < 0.001) and nurses’ perceptions of nurse managers’ authentic leadership (B = 0.142, *p* = 0.009) were also positively associated with nursing performance, as was teamwork (B = 0.145, *p* = 0.023). The indirect effect through teamwork was 0.029 for communication competence (95% CI [−0.003, 0.085]) and 0.071 for nurses’ perceptions of nurse managers’ authentic leadership (95% CI [−0.007, 0.159]). Because both confidence intervals included zero, neither indirect association was statistically significant.

To examine potential measurement overlap, discriminant validity was assessed between the predictor measures and the corresponding T-TPQ subscales. Communication competence reflects individual nurses’ interpersonal communication abilities [38], whereas the T-TPQ communication subscale assesses communication processes among team members [42]. Consistent with this conceptual distinction, the two measures showed limited overlap (HTMT = 0.22; latent factor correlation = 0.37, 95% CI [0.16, 0.58]). In contrast, both nurses’ perceptions of nurse managers’ authentic leadership and the T-TPQ leadership subscale were based on nurses’ evaluations of the same nurse manager, although they assessed different aspects of leadership. These measures showed a relatively strong association (HTMT = 0.80; latent factor correlation = 0.83, 95% CI [0.74, 0.91]), suggesting potential measurement overlap. Accordingly, the T-TPQ leadership subscale was excluded from the modified teamwork score in a further sensitivity analysis, whereas the communication subscale was retained.

A further sensitivity analysis was therefore conducted using a modified 28-item teamwork score that excluded the seven-item T-TPQ leadership subscale, while retaining both predictors in the same model. Communication competence (B = 0.244, *p* = 0.007) and nurses’ perceptions of nurse managers’ authentic leadership (B = 0.412, *p* < 0.001) remained positively associated with teamwork. Their direct associations with nursing performance were also significant (B = 0.239, *p* < 0.001, and B = 0.140, *p* = 0.005, respectively), as was the association between teamwork and nursing performance (B = 0.176, *p* = 0.002). In this modified model, the indirect effect through teamwork was 0.043 for communication competence (95% CI [0.004, 0.107]) and 0.073 for nurses’ perceptions of nurse managers’ authentic leadership (95% CI [0.018, 0.139]). Because both confidence intervals excluded zero, both indirect associations were statistically significant.

An additional sensitivity analysis was conducted using a modified 21-item teamwork score that excluded both the leadership and communication subscales of the T-TPQ (Supplementary Table S1). In this mutually adjusted model, the indirect effect through teamwork was 0.047 for communication competence (95% CI [0.007, 0.109]) and 0.071 for nurses’ perceptions of nurse managers’ authentic leadership (95% CI [0.020, 0.131]). Because both confidence intervals excluded zero, both indirect associations were statistically significant.

Monte Carlo power analyses based on the observed standardized path coefficients yielded estimated powers of 0.99 and 0.88 for the indirect effects of communication competence and nurses’ perceptions of nurse managers’ authentic leadership, respectively, in the separate models. The corresponding estimates were 0.37 and 0.63 in the mutually adjusted models using the full 35-item teamwork score, and 0.59 and 0.86 in those using the 28-item teamwork score.

### 3.6. Overall Assessment of Hypotheses

The results of the separate mediation models supported Hypotheses 1–7 (Table 4). The positive associations of communication competence and nurses’ perceptions of nurse managers’ authentic leadership with teamwork and nursing performance, as well as the association between teamwork and nursing performance, were also observed in the mutually adjusted models (Table 5 and Supplementary Table S1). However, support for the hypothesized indirect associations (Hypotheses 4 and 7) varied according to model specification. Both indirect associations were statistically significant in the separate models, but neither was statistically significant in the mutually adjusted model using the full 35-item teamwork score. In contrast, both indirect associations were statistically significant in the mutually adjusted models using the 28-item and 21-item teamwork scores. Overall, Hypotheses 1–3, 5, and 6 were supported, whereas support for Hypotheses 4 and 7 was dependent on model specification.

## 4. Discussion

This study used McGrath’s IPO model [30] as its theoretical framework to examine whether OR nurses’ communication competence and nurses’ perceptions of nurse managers’ authentic leadership are associated with nursing performance and whether these associations involve indirect associations through teamwork. In the two separately estimated mediation models, both predictors showed significant positive associations with teamwork and nursing performance, as well as significant indirect associations with nursing performance through teamwork. However, neither indirect association remained statistically significant in the mutually adjusted model using the full teamwork measure. When the leadership subscale was excluded from the teamwork measure, both indirect associations were statistically significant in the mutually adjusted sensitivity analysis. Both indirect associations also remained statistically significant in the additional 21-item sensitivity analysis excluding the leadership and communication subscales. These findings indicate that the observed indirect associations varied according to model specification and the measurement of teamwork.

In this study, the mean nursing performance score among OR nurses was 3.30 out of 4, compared with scores of 3.23 [45] and 3.08 [10] reported in previous studies using the same instrument. However, these differences are descriptive and should not be interpreted as evidence of higher nursing performance, as differences in participant characteristics and clinical settings across studies may have influenced the observed scores. Nursing performance also differed significantly according to education level, job position, and total clinical experience. Higher scores were observed among nurses with a master’s degree or higher, charge nurses, and those with at least five years of total clinical experience. These findings are consistent with previous studies of OR nurses reporting differences in nursing performance according to education level, job position, and clinical experience [9,46]. Similar findings regarding education level and clinical experience have also been reported among clinical nurses [48]. Taken together, these findings suggest that educational preparation, accumulated clinical experience, and differences in professional responsibilities may be relevant to nursing performance among OR nurses.

Communication competence among OR nurses showed significant positive associations with both teamwork and nursing performance, and teamwork was likewise positively associated with nursing performance, supporting Hypotheses 1–3. The positive association between communication competence and teamwork aligns with the findings of a recent study conducted among Korean OR nurses, which reported a significant positive correlation between these variables [16]. Similarly, the association between nurses’ communication competence and nursing performance is consistent with the findings of previous studies concerning staff nurses [14,15]. Furthermore, Dlamini and Park [49] reported positive associations between communication skills and teamwork, as well as their associations with patient-centered care. Although studies directly examining the association between teamwork and nursing performance among OR nurses are limited, inter-nurse and physician–nurse collaboration among OR nurses has been found to be positively associated with nursing performance [9]. A recent study of OR nurses also reported a positive association between team psychological safety and job performance, highlighting the relevance of team-related factors to nursing performance [8]. Teamwork has also been linked to patient safety management activities [26] and adherence to surgical patient safety protocols [27], and surgical outcomes [28]. Taken together, these findings suggest that understanding nursing performance among OR nurses requires the consideration of both individual communication competence and collaborative interactions among team members.

Furthermore, a significant indirect association through teamwork was found in the separate mediation model for the association between communication competence and nursing performance. This finding suggests that communication competence may be related to nursing performance both directly and indirectly through teamwork. A study of nursing teams reported an indirect association through teamwork between team members’ role characteristics and team performance [33]. Similarly, in multidisciplinary healthcare teams, teamwork partially mediated the association between knowledge integration and team performance [50]. Although these studies differed from the present study in the nature of the input factors and levels of analysis, they are theoretically consistent with the IPO framework used here, in which teamwork is conceptualized as a process through which communication competence may be associated with nursing performance. In the perioperative setting, Marrone [51] reported that an interprofessional collaboration initiative was associated with reduced operating room turnover times and increased staff satisfaction, suggesting the relevance of collaborative team processes to surgical team performance. However, the indirect association observed in the separate model was no longer statistically significant when nurses’ perceptions of nurse managers’ authentic leadership were included as an additional predictor, in the model using the full 35-item teamwork score. In contrast, the indirect association was statistically significant in the mutually adjusted 28-item and 21-item models, although the estimated indirect effects were smaller than in the separate model. These findings indicate that the indirect association was sensitive to model specification and the measurement of teamwork.

OR nurses’ perceptions of nurse managers’ authentic leadership were positively associated with teamwork and remained significantly associated with nursing performance after accounting for teamwork in the separate mediation model, supporting Hypotheses 5 and 6. The positive association between nurses’ perceptions of nurse managers’ authentic leadership and teamwork aligns with previous findings that nurse managers’ authentic leadership is significantly related to nurses’ teamwork competence and interprofessional collaboration [19,20]. Similarly, the association between nurses’ perceptions of nurse managers’ authentic leadership and nursing performance is consistent with findings from an earlier study reporting a relationship between nurses’ perceptions of nurse managers’ authentic leadership and job performance [21], as well as a recent study reporting an association with quality of care [22]. These findings highlight the relevance of nurses’ perceptions of nurse managers’ authentic leadership to understanding nursing performance in the OR, alongside individual competencies and teamwork.

Furthermore, a significant indirect association through teamwork was found in the separate mediation model for the association between nurses’ perceptions of nurse managers’ authentic leadership and nursing performance. This finding suggests that nursing performance may be related to nurses’ perceptions of nurse managers’ authentic leadership both directly and indirectly through teamwork. Although previous research has linked nurse managers’ authentic leadership to nursing team performance [52], studies directly examining whether teamwork mediates the association between authentic leadership and nursing performance remain limited. However, research involving hospital nurses has shown a significant mediating effect of teamwork in the association between authentic leadership and work engagement [53], providing related evidence for considering teamwork as a process associated with authentic leadership and nurses’ work-related outcomes. Other studies have also reported positive associations among authentic leadership, team effectiveness, and satisfaction with the quality of care [54], as well as an indirect association between authentic leadership and affective and normative organizational commitment through workplace social capital [55]. Although team effectiveness and workplace social capital are conceptually distinct from teamwork, these findings further support the relevance of team-related and relational factors to the associations between authentic leadership and nurses’ work-related outcomes. Nevertheless, the indirect association observed in the separate model was no longer statistically significant when communication competence was included as an additional predictor in the model using the full 35-item teamwork measure. In contrast, the indirect association was statistically significant in the mutually adjusted 28-item and 21-item models, although the estimated indirect effects were smaller than in the separate model. These findings indicate that the statistical significance of the indirect association varied across model specifications and warrants cautious interpretation.

Taken together, the two separate mediation models showed that two conceptually distinct factors, nurses’ communication competence as an individual-level input and nurses’ perceptions of nurse managers’ authentic leadership as a leadership-related input, were each positively associated with nursing performance, with significant indirect associations through teamwork in both models. Notably, the direct associations of both predictors with nursing performance remained statistically significant across all model specifications. However, neither indirect association remained statistically significant when both predictors were included simultaneously in the model using the full 35-item teamwork measure. In contrast, both indirect associations were statistically significant in the mutually adjusted model using the modified 28-item teamwork measure that excluded the T-TPQ leadership subscale. Both indirect associations also remained statistically significant in the additional 21-item model excluding the leadership and communication subscales. However, the estimated indirect effects in the mutually adjusted 28-item and 21-item models were smaller than those in the separate models. In interpreting these differences, it is important to note that nurses rated the same nurse manager on both authentic leadership and the T-TPQ leadership subscale, and the relatively strong association between these measures may partly reflect overlap in leadership-related content. In contrast, the limited overlap between communication competence and the T-TPQ communication subscale supported their conceptual distinction. The potential overlap in leadership-related content may have contributed to the differences observed across the sensitivity analyses. However, attributing these differences solely to potential measurement overlap would be premature, as excluding the leadership subscale also changed the composition of the teamwork measure. Furthermore, the observed-path Monte Carlo power estimates were lower in the mutually adjusted models, particularly for communication competence, indicating that the indirect effect estimates warrant cautious interpretation. The non-significant findings in the 35-item model should therefore not be interpreted as evidence of the absence of indirect associations, while the statistically significant findings in the modified models should also be considered in light of the uncertainty in their estimates.

The indirect effects observed in the separate mediation models were statistically significant (B = 0.103 and 0.106; completely standardized indirect effects = 0.122 and 0.172, respectively). These standardized estimates suggest that the indirect associations through teamwork were relatively modest in magnitude. In the mutually adjusted 28-item model, the unstandardized indirect effects were smaller (B = 0.043 and 0.073), corresponding to estimated differences of 0.043 and 0.073 points in nursing performance, respectively, per one-point difference in each predictor through teamwork. These estimates provide a measure of the magnitude of the indirect associations, although their practical importance cannot be determined from the statistical estimates alone. From a practical perspective, these findings highlight the importance of considering teamwork alongside individual communication competence and nurse managers’ authentic leadership, rather than expecting teamwork alone to account for substantial differences in nursing performance. The observed associations provide an integrated perspective in which nurses’ communication competence and their perceptions of nurse managers’ authentic leadership are considered alongside perceived teamwork and nursing performance in the highly interdependent OR context. These findings are consistent with the conceptual relationships proposed in McGrath’s IPO model but do not establish a causal input–process–output mechanism.

### 4.1. Implications of the Findings

The findings of this study suggest the need to consider individual-, manager-, and team-focused approaches when developing strategies to support nursing performance among OR nurses. At the individual level, education aimed at strengthening OR nurses’ communication competence may be considered, while at the manager level, the development of authentic leadership competencies among nurse managers may be supported. For surgical teams, strategies to strengthen communication and teamwork among surgical team members may also be considered. Given the variation in the indirect associations across sensitivity analyses and the cross-sectional design, these findings provide a basis for further investigating, rather than establishing the effectiveness of, individual-, manager-, and team-focused approaches. Future longitudinal studies should clarify the temporal relationships among these factors, while intervention studies should evaluate the effectiveness of programs integrating individual-, manager-, and team-focused components.

### 4.2. Strengths and Limitations

One strength of this study is that it applied McGrath’s IPO model [30] to integrate conceptually distinct individual- and leadership-related factors within a common teamwork process. Specifically, nurses’ communication competence and their perceptions of nurse managers’ authentic leadership were examined as separate input factors, and their direct and indirect associations with nursing performance through teamwork were tested in two separate mediation models. Additional sensitivity analyses examined these associations after mutual adjustment of the predictors and modification of the teamwork measure. This approach provides a more integrated understanding of how individual competence and leadership-related factors may be linked to nursing performance in the highly interdependent OR context.

However, this study has some limitations. First, the cross-sectional design precludes establishing temporal relationships among variables, and the observed indirect associations should not be interpreted as evidence of causal mediation. Second, all main variables, including nursing performance, were self-reported, raising concerns about common method bias. Although additional statistical assessments were conducted, Harman’s single-factor test has limited sensitivity for detecting common method bias [56], and residual measurement-related bias cannot be excluded. Furthermore, despite voluntary recruitment and confidentiality procedures, potential participation pressure or socially desirable responses related to evaluating nurse managers cannot be entirely ruled out. Potential measurement overlap between authentic leadership and the T-TPQ leadership subscale should also be considered. In addition, teamwork and nursing performance were assessed through individual nurses’ perceptions rather than at the team level or through objective performance measures, respectively.

Third, the nonprobability sample from three tertiary hospitals limits generalizability. Hospital- and manager-level clustering could not be accounted for because the relevant identifiers were unavailable for analysis, potentially affecting the precision of the estimates. Finally, sample size estimation was based on multiple regression rather than the statistical power of indirect effects, leaving uncertainty regarding the detection of smaller indirect associations. Future studies using longitudinal designs, independent performance assessments, and appropriately powered multilevel analyses may help address these limitations.

## 5. Conclusions

In this study, OR nurses’ communication competence and their perceptions of nurse managers’ authentic leadership were each significantly and positively associated with teamwork and nursing performance. Their direct associations with nursing performance remained statistically significant across all model specifications. In contrast, the indirect associations through teamwork were sensitive to model specification and the measurement of teamwork, warranting cautious interpretation. Nevertheless, these findings suggest that understanding factors associated with nursing performance among OR nurses requires considering teamwork as a team interaction process perceived by individual nurses, alongside individual communication competence and nurses’ perceptions of nurse managers’ authentic leadership. Given the cross-sectional design, these findings reflect statistical associations rather than causal relationships and should not be interpreted as evidence that improving communication competence, authentic leadership, or teamwork would necessarily improve nursing performance. Future longitudinal studies should clarify the temporal relationships among these factors, while intervention studies should evaluate strategies integrating individual-, manager-, and team-focused approaches.

## Figures and Tables

**Table 1:** Participants’ demographic and work-related characteristics and differences in nursing performance (N = 149).

Characteristics	Categories	n	%	Mean ± SD	t or F	*p*
Gender	Male	19	12.8	3.31 ± 0.33	0.067	0.946
	Female	130	87.2	3.30 ± 0.34		
Age (years)	≤25	16	10.8	3.30 ± 0.34	0.271	0.846
	26–30	55	36.9	3.27 ± 0.29		
	31–35	37	24.8	3.33 ± 0.41		
	≥36	41	27.5	3.32 ± 0.33		
	Mean ± SD	32.93 ± 7.35				
Marital status	Single	96	64.4	3.30 ± 0.35	−0.215	0.830
	Married	53	35.6	3.31 ± 0.32		
Religion	Yes	44	29.5	3.35 ± 0.33	1.046	0.297
	No	105	70.5	3.28 ± 0.34		
Education level	Diploma ^a^	20	13.4	3.21 ± 0.28	3.224	0.043
	Bachelor’s degree ^b^	104	69.8	3.29 ± 0.33		a, b < c *
	Master’s degree or higher ^c^	25	16.8	3.44 ± 0.37		
Job position	Staff nurse	93	62.4	3.24 ± 0.32	−2.738	0.007
	Charge nurse	56	37.6	3.40 ± 0.35		
Total clinical experience (years)	1–<5 ^a^	46	30.8	3.19 ± 0.31	4.140	0.018
	5–<10 ^b^	50	33.6	3.36 ± 0.35		a < b, c *
	≥10 ^c^	53	35.6	3.35 ± 0.33		
	Mean ± SD	9.50 ± 7.08				
Perioperative nursing experience (years)	1–<5	49	32.9	3.21 ± 0.32	2.991	0.053
	5–<10	49	32.9	3.34 ± 0.28		
	≥10	51	34.2	3.34 ± 0.35		
	Mean ± SD	9.01 ± 6.94				
Work schedule	Fixed	16	10.7	3.35 ± 0.33	0.874	0.384
	Shift	133	89.3	3.29 ± 0.33		
Reasons for operating room assignment	Voluntary	106	71.1	3.31 ± 0.35	0.613	0.541
	Assigned	43	28.9	3.27 ± 0.30		
Job satisfaction	Satisfied	70	47.0	3.37 ± 0.32	2.515	0.084
	Neutral	69	46.3	3.24 ± 0.35		
	Dissatisfied	10	6.7	3.29 ± 0.34		

SD: Standard deviation; * Post hocScheffé test. Superscript letters (a, b, and c) indicate the groups used for post hoc comparisons.

**Table 2:** Levels of the main study variables (N = 149).

Variable	Mean ± SD	Min	Max	Skewness	Kurtosis
Communication competence	3.81 ± 0.40	2.67	4.93	0.068	0.383
Nurse managers’ authentic leadership	3.86 ± 0.55	2.06	5.00	0.211	−0.240
Self-awareness	3.92 ± 0.60	2.25	5.00	−0.093	0.496
Relational transparency	3.90 ± 0.59	1.60	5.00	−0.121	−0.301
Internalized moral perspective	3.85 ± 0.67	1.75	5.00	0.048	−0.454
Balanced processing	3.72 ± 0.68	1.67	5.00	0.016	0.230
Teamwork	3.80 ± 0.49	2.60	5.00	0.215	−0.498
Team structure	3.87 ± 0.57	1.86	5.00	−0.019	0.378
Leadership	3.58 ± 0.64	1.86	5.00	0.016	−0.113
Situation monitoring	3.95 ± 0.58	2.29	5.00	−0.217	0.782
Mutual support	3.84 ± 0.53	2.29	5.00	−0.273	0.210
Communication	3.75 ± 0.60	2.57	5.00	−0.342	0.336
Nursing performance	3.30 ± 0.34	2.53	4.00	0.373	−0.775
Performance capability	3.37 ± 0.37	2.71	4.00	0.230	−1.259
Performance attitude	3.22 ± 0.44	2.25	4.00	0.287	−0.613
Performance improvement	3.33 ± 0.45	2.67	4.00	0.367	−1.228
Nursing process application	3.23 ± 0.52	2.00	4.00	0.098	−0.547

Note. SD: Standard deviation. Nurse managers’ authentic leadership refers to nurses’ perceptions of nurse managers’ authentic leadership.

**Table 3:** Correlations among communication competence, nurses’ perceptions of nurse managers’ authentic leadership, teamwork, and nursing performance (N = 149).

Variables	Communication Competence	Nurse Managers’ Authentic Leadership	Teamwork	Nursing Performance
	r (*p*)	r (*p*)	r (*p*)	r (*p*)
Communication competence	1			
Nurse managers’ authentic leadership	0.332 (<0.001)	1		
Teamwork	0.340 (<0.001)	0.650 (<0.001)	1	
Nursing performance	0.451 (<0.001)	0.393 (<0.001)	0.396 (<0.001)	1

Note. Nurse managers’ authentic leadership refers to nurses’ perceptions of nurse managers’ authentic leadership.

**Table 4:** Direct and indirect associations of communication competence and nurses’ perceptions of nurse managers’ authentic leadership with nursing performance through teamwork.

Path/Effect	B	SE	β	t	*p*	95% CI [LL, UL]
Model 1: Communication competence → Teamwork → Nursing performance						
Communication competence → Teamwork (a)	0.428	0.094	0.349	4.533	<0.001	0.241, 0.614
Communication competence → Nursing performance (c′)	0.277	0.063	0.327	4.394	<0.001	0.152, 0.402
Teamwork → Nursing performance (b)	0.241	0.052	0.349	4.599	<0.001	0.137, 0.344
Total effect (c)	0.380	0.063	0.449	6.036	<0.001	0.256, 0.505
Indirect effect through teamwork (a × b)	0.103	0.038 *				0.042 *, 0.191 *
Completely standardized indirect effect through teamwork	0.122					0.051 *, 0.222 *
Model 2: Nurse managers’ authentic leadership → Teamwork → Nursing performance						
Nurse managers’ authentic leadership → Teamwork (a)	0.538	0.056	0.605	9.564	<0.001	0.427, 0.649
Nurse managers’ authentic leadership → Nursing performance (c‘)	0.174	0.056	0.283	3.121	0.002	0.064, 0.284
Teamwork → Nursing performance (b)	0.196	0.065	0.284	3.031	0.003	0.068, 0.324
Total effect (c)	0.279	0.045	0.455	6.257	<0.001	0.191, 0.367
Indirect effect through teamwork (a × b)	0.106	0.048 *				0.022 *, 0.209 *
Completely standardized indirect effect through teamwork	0.172					0.036 *, 0.335 *

Note. B = Unstandardized coefficient; SE = Standard error; β = Standardized regression coefficient; CI = Confidence interval; LL = Lower limit; UL = Upper limit. Path a denotes the association between the independent variable and teamwork; path b, the association between teamwork and nursing performance adjusted for the independent variable; path c′, the direct association adjusted for teamwork; and path c, the total association between the independent variable and nursing performance. Both models were adjusted for education level, job position, and total clinical experience. * Bootstrap standard error and 95% percentile bootstrap CI based on 5000 bootstrap samples. Nurse managers’ authentic leadership refers to nurses’ perceptions of nurse managers’ authentic leadership.

**Table 5:** Sensitivity analyses of direct and indirect associations with nursing performance through teamwork in mutually adjusted models.

Path/Effect	B	SE	β	t	*p*	95% CI [LL, UL]
Model 1: Mutually Adjusted Model Using the Full T-TPQ (35 items)						
Communication competence → Teamwork	0.202	0.082	0.164	2.461	0.015	0.040, 0.364
Nurse managers’ authentic leadership → Teamwork	0.490	0.059	0.551	8.380	<0.001	0.375, 0.606
Communication competence → Nursing performance	0.253	0.062	0.298	4.050	<0.001	0.129, 0.376
Nurse managers’ authentic leadership → Nursing performance	0.142	0.053	0.231	2.659	0.009	0.036, 0.248
Teamwork → Nursing performance	0.145	0.063	0.210	2.304	0.023	0.021, 0.269
Indirect effect of communication competence through teamwork	0.029	0.023 *				−0.003 *, 0.085 *
Indirect effect of authentic leadership through teamwork	0.071	0.042 *				−0.007 *, 0.159 *
Model 2: Mutually Adjusted Model Excluding the T-TPQ Leadership Subscale (28 items)						
Communication competence → Teamwork	0.244	0.090	0.195	2.718	0.007	0.067, 0.421
Nurse managers’ authentic leadership → Teamwork	0.412	0.064	0.456	6.433	<0.001	0.286, 0.539
Communication competence → Nursing performance	0.239	0.062	0.282	3.869	<0.001	0.117, 0.361
Nurse managers’ authentic leadership → Nursing performance	0.140	0.049	0.229	2.871	0.005	0.044, 0.237
Teamwork → Nursing performance	0.176	0.056	0.259	3.118	0.002	0.064, 0.288
Indirect effect of communication competence through teamwork	0.043	0.027 *				0.004 *, 0.107 *
Indirect effect of authentic leadership through teamwork	0.073	0.031 *				0.018 *, 0.139 *

Note. B = Unstandardized coefficient; SE = Standard error; β = Standardized regression coefficient; CI = Confidence interval; LL = Lower limit; UL = Upper limit. Both models simultaneously included communication competence and nurses’ perceptions of nurse managers’ authentic leadership and were adjusted for education level, job position, and total clinical experience. Model 1 used the full 35-item T-TPQ, whereas Model 2 used the 28-item teamwork score excluding the leadership subscale. * Bootstrap standard errors and 95% percentile bootstrap CIs were estimated using 5000 bootstrap samples. Nurse managers’ authentic leadership refers to nurses’ perceptions of nurse managers’ authentic leadership.

## Data Availability

The datasets used and/or analyzed during the current study are available from the corresponding author on reasonable request, subject to ethical and institutional restrictions and the protection of participants’ confidentiality.

## Supplementary Materials

The following supporting information can be downloaded at: https://www.mdpi.com/article/10.3390/healthcare14213706/s1, Table S1: Sensitivity analysis of direct and indirect associations with nursing performance through teamwork in the mutually adjusted model excluding the T-TPQ leadership and communication subscales (21 items). Table S2: STROBE Statement—Checklist of items that should be included in reports of cross-sectional studies.
