# Supplementary material for: Communication Competence and Nurse Managers’ Authentic Leadership in Relation to Nursing Performance Among Operating Room Nurses: The Role of Teamwork in a Cross-Sectional Study
Source: Healthcare (Basel). 2026 Nov 1;14(21):3706. doi: 10.3390/healthcare14213706 (PMC13763369; doi:10.3390/healthcare14213706)
Supplement: Supplementary file 1 [file healthcare-14-03706-s001.zip › healthcare-4619272-supplementary.pdf]

Supplementary Material

Manuscript ID: healthcare-4619272 Communication Competence and Nurse Managers' Authentic Leadership in Relation to Nursing Performance Among Operating Room Nurses: The Role of Teamwork in a Cross-Sectional Study

Table S1. Sensitivity analysis of direct and indirect associations with nursing performance through teamwork in the mutually adjusted model excluding the T-TPQ leadership and communication subscales (21 items).

**Path/effect**	**B**	**SE**	**β**	**t**	**p**	**95% CI [LL, UL]**
Communication competence → Teamwork	0.269	0.095	0.206	2.823	0.005	0.081, 0.457
Nurse managers' authentic leadership → Teamwork	0.405	0.068	0.428	5.956	< 0.001	0.270, 0.539
Communication competence → Nursing performance	0.235	0.062	0.277	3.810	< 0.001	0.113, 0.357
Nurse managers' authentic leadership → Nursing performance	0.142	0.048	0.232	2.966	0.004	0.047, 0.237
Teamwork → Nursing performance	0.175	0.053	0.270	3.330	0.001	0.070, 0.280
Indirect effect of communication competence through teamwork	0.047	0.026*				0.007*, 0.109*
Indirect effect of authentic leadership through teamwork	0.071	0.028*				0.020*, 0.131*

Note. B = Unstandardized coefficient; SE = Standard error; β = Standardized regression coefficient; CI = Confidence interval; LL = Lower limit; UL = Upper limit. The model simultaneously included communication competence and nurses' perceptions of nurse managers' authentic leadership and was adjusted for education level, job position, and total clinical experience. Teamwork was assessed using the 21-item T-TPQ score excluding the leadership and communication subscales. *Bootstrap standard errors and 95% percentile bootstrap CIs were estimated using 5,000 bootstrap samples. Nurse managers' authentic leadership refers to nurses' perceptions of nurse managers' authentic leadership.

Table S2. STROBE Statement—Checklist of items that should be included in reports of cross-sectional studies

Item No.	Recommendation	Location in the Manuscript
Title and abstract		
1a	Indicate the study's design with a commonly used term in the title or the abstract.	Title, p. 1; Abstract, pp. 1–2
1b	Provide in the abstract an informative and balanced summary of what was done and what was found.	Abstract, pp. 1–2
Introduction		
2	Explain the scientific background and rationale for the investigation being reported.	Section 1, pp. 2–3
3	State specific objectives, including any prespecified hypotheses.	Section 1, p. 3; Section 1.2, pp. 3–4
Methods		
4	Present key elements of study design early in the paper.	Section 2.1, p. 4
5	Describe the setting, locations, and relevant dates, including periods of recruitment, exposure, follow-up, and data collection.	Section 2.1, p. 4; Section 2.4, p. 6; exposure/follow-up: Not applicable
6a	Give the eligibility criteria, and the sources and methods of selection of participants.	Section 2.2, pp. 4–5; Section 2.4, p. 6
7	Clearly define all outcomes, exposures, predictors, potential confounders, and effect modifiers. Give diagnostic criteria, if applicable.	Section 1.1 and 1.2, pp. 3–4; Sections 2.3 and 2.5, pp. 5–7
8	For each variable of interest, give sources of data and details of methods of assessment (measurement). Describe comparability of assessment methods if there is more than one group.	Sections 2.3.1–2.3.5, pp. 5–6; Section 2.4, p. 6
9	Describe any efforts to address potential sources of bias.	Sections 2.4–2.5, pp. 6–7
10	Explain how the study size was arrived at.	Section 2.2, p. 4–5
11	Explain how quantitative variables were handled in the analyses. If applicable, describe which groupings were chosen and why.	Sections 2.3 and 2.5, pp. 5–7; Table 1, pp. 8–9
12a	Describe all statistical methods, including those used to control for confounding.	Section 2.5, pp. 6–7; Tables 4–5, pp. 10–13
12b	Describe any methods used to examine subgroups and interactions.	Not applicable (no subgroup or interaction analyses reported)
12c	Explain how missing data were addressed.	Section 2.2, p. 5
12d	If applicable, describe analytical methods taking account of sampling strategy.	Not applicable (no design-based adjustment)
12e	Describe any sensitivity analyses.	Section 2.5, pp. 6–7; Section 3.5, pp. 11–13; Table 5, pp. 12–13
Results		
13a	Report numbers of individuals at each stage of study—e.g., numbers potentially eligible, examined for eligibility, confirmed eligible, included in the study, completing follow-up, and analysed.	Section 2.2, p. 5
13b	Give reasons for non-participation at each stage.	Section 2.2, p. 5
13c	Consider use of a flow diagram.	No flow diagram; Section 2.2, p. 5
14a	Give characteristics of study participants (e.g., demographic, clinical, social) and information on exposures and potential confounders.	Section 3.1 and Table 1, pp. 8–9
14b	Indicate number of participants with missing data for each variable of interest.	Section 2.2, p. 5
15	Report numbers of outcome events or summary measures.	Sections 3.1–3.3 and Tables 1–3, pp. 8–10
16a	Give unadjusted estimates and, if applicable, confounder-adjusted estimates and their precision (e.g., 95% confidence interval). Make clear which confounders were adjusted for and why they were included.	Section 2.5, pp. 6–7; Sections 3.3–3.5 and Tables 3–5, pp. 10–13
16b	Report category boundaries when continuous variables were categorised.	Section 2.5, p. 7; Table 1, pp. 8–9
16c	If relevant, consider translating estimates of relative risk into absolute risk for a meaningful time period.	Not applicable (no relative-risk estimates)
17	Report other analyses done—e.g., analyses of subgroups and interactions, and sensitivity analyses.	Section 2.5, pp. 6–7; Section 3.5 and Table 5, pp. 11–13
Discussion		
18	Summarise key results with reference to study objectives.	Sections 3.6 and 4, pp. 13–17; Section 5, p. 17

19	Discuss limitations of the study, taking into account sources of potential bias or imprecision. Discuss both direction and magnitude of any potential bias.	Section 4.2, pp. 16–17
20	Give a cautious overall interpretation of results considering objectives, limitations, multiplicity of analyses, results from similar studies, and other relevant evidence.	Sections 3.6 and 4–4.2, pp. 13–17; Section 5, p. 17
21	Discuss the generalisability (external validity) of the study results.	Section 4.2, pp. 16–17
Other information		
22	Give the source of funding and the role of the funders for the present study and, if applicable, for the original study on which the present article is based.	Funding and Conflicts of Interest, pp. 17–18

Note. Section and page references correspond to the revised manuscript. “Not applicable” indicates items that do not apply to the present study. STROBE = Strengthening the Reporting of Observational Studies in Epidemiology.
